# Lipid-Laden Multilocular Cells in the Aging Thymus Are Phenotypically Heterogeneous

**DOI:** 10.1371/journal.pone.0141516

**Published:** 2015-10-28

**Authors:** Larissa G. P. Langhi, Leonardo R. Andrade, Marilia Kimie Shimabukuro, Willem van Ewijk, Dennis D. Taub, Radovan Borojevic, Valeria de Mello Coelho

**Affiliations:** 1 Institute of Biomedical Sciences, Health Sciences Center, Federal University of Rio de Janeiro, Rio de Janeiro, Brazil; 2 Department of Immunology, Erasmus University, Rotterdam, Netherlands; 3 Department of Veterans Affairs, Medical Services, Veterans Affairs Medical Center, Washington, District of Columbia, United States of America; University of Tokyo, JAPAN

## Abstract

Intrathymic lipid-laden multilocular cells (LLMC) are known to express pro-inflammatory factors that might regulate functional activity of the thymus. However, the phenotype of age-associated intrathymic LLMC is still controversial. In this study, we evaluated LLMC density in the aging thymus and better characterized their distribution, ultrastructure and phenotype. Our results show an increased density of LLMC in the thymus from 03 to 24 months of age. Morphologically, intrathymic LLMC exhibit fibroblastoid fusiform, globular or stellate shapes and can be found in the subcapsular region as well as deeper in the parenchyma, including the perivascular area. Some parenchymal LLMC were like telocytes accumulating lipids. We identified lipid droplets with different electrondensities, lipofuscin granules and autolipophagosome-like structures, indicating heterogeneous lipid content in these cells. Autophagosome formation in intrathymic LLMC was confirmed by positive staining for beclin-1 and perilipin (PLIN), marker for lipid droplet-associated proteins. We also found LLMC in close apposition to thymic stromal cells, endothelial cells, mast cells and lymphocytes. Phenotypically, we identified intrathymic LLMC as preadipocytes (PLIN^+^PPARγ2^+^), brown adipocytes (PLIN^+^UCP1^+^), macrophages (PLIN^+^Iba-1^+^) or pericytes (PLIN^+^NG2^+^) but not epithelial cells (PLIN^-^ panCK^+^). These data indicate that intrathymic LLMC are already present in the young thymus and their density significantly increases with age. We also suggest that LLMC, which are morphologically distinct, establish direct contact with lymphocytes and interact with stromal cells. Finally, we evidence that intrathymic LLMC correspond to not only one but to distinct cell types accumulating lipids.

## Introduction

The thymus, a primary lymphoid organ responsible for T cell maturation, decreases its functionality with age [1]. This organ is bi-lobed and subdivided in lobules by septa that emerge from the capsule of connective tissue [1–2]. To enter in the thymic parenchyma, T lymphocyte progenitors, originated in the bone marrow, need to pass through the thymic-blood barrier, which is formed by endothelial cells and the perivascular space rich in extracellular matrix surrounded by parenchymal epithelial cells [3]. Once inside the thymic parenchyma, T lymphocyte progenitors interact with stromal cells, including thymic epithelial cells, which support their differentiation and migration through the production of thymic hormones, chemokines and several growth factors [2,4–6]. Following the T cell receptor gene rearrangement, T lymphocytes are able of recognizing self-peptides-loaded MHC molecules expressed on thymic stromal cells. However, when this recognition does not occur with the proper avidity, thymocytes undergo apoptosis, which occurs in ~95% of T cells during differentiation. The apoptotic thymic T cells are cleared through engulfment by macrophages [7]. Thymocytes also interact with dendritic cells, which act in central tolerance, stimulating the elimination of self-reactive T cell clones and the generation of regulatory thymic T cells [8]. Thymic epithelial cells participate in the process of T cell tolerance as well [1].

With age, the thymus suffers changes in its architecture, losing a clear demarcation between cortex and medullary regions, which is related to lymphocyte cell death [1,9]. Other changes observed in the aging thymus include a decrease in the number and activity of thymic epithelial cells, reducing growth factors production [1, 10–11]. Moreover, there is a progressive increase of adipose tissue deposition in the capsule and septa regions [10]. Mature adipocytes in the thymic perivascular space are morphologically recognized for the presence of large lipid droplets or a large lipid locule in the cytoplasm [12] and the role of these cells in the thymus is still poorly known. Sempowsky *et al*. showed that there is an increase in the production of mRNA for gp130 ligands, including IL-6, LIF and Oncostatin-M in the human thymus with age progression [10]. In this context, human adipose tissue cells produce pro-inflammatory cytokines, including ligands for gp130 receptor [10].

Adipocytes in differentiation are morphologically distinct of mature adipocytes due to the presence of numerous lipid droplets in their cytoplasm before becoming unilocular mature cells [12, 13], characterizing lipid-laden multilocular cells (LLMC). The presence and origin of LLMC inside the thymic parenchyma (not only in adipose tissue in the capsule and septa) is still not well known. To our knowledge, the first report on intrathymic LLMC was in 1952, when they were described as lipid foam cells, in old rats, based on their similarity with cells found in fat degeneration such as hepatic steatosis [14]. Later, Spicer mentioned (but did not show) thymic lipid-laden phagocytes interacting with mast cells [15]. Mast cells are known to have participation in inflammatory processes [16]. Functionally, we have previously shown that intrathymic LLMC express pro-inflammatory factors, including LIF and CCR5 chemokine receptor in aged mice [17–18]. We also suggested that intrathymic LLMC could correspond to preadipocytes [17]. Other groups suggested that intrathymic LLMC in the aging thymus could correspond to parenchymal thymic epithelial cells transdifferentiated to mesenchymal cells able of accumulating lipids [19, 20]. Conjointly, these studies show that the phenotype and origin of intrathymic LLMC are still controversial in the literature. Herein, we sought to evaluate changes in intrathymic LLMC density with age and better characterize their distribution, morphology, including ultrastructure, and their phenotype. Our results indicate that intrathymic LLMC density significantly increases with age and clearly show that these cells may establish membrane contacts with lymphocytes or interact with stromal cells. Finally, we identify intrathymic LLMC corresponding to not only one but to distinct cell types, including professional and non-professional lipid laden cells.

## Materials and Methods

### Animals

Female Balb/c mice, age ranging between 02 and 24 months, were kept under a constant light-dark cycle (12h) and temperature of 25°C, with standard food and water access *ad libitum*. Mice were euthanized with CO_2_ or anesthetized with ether for transcardial perfusion. All procedures were performed under the approval of the Committee of Ethics in Use of Animals of the Federal University of Rio de Janeiro.

### Transmission electron microscopy (TEM)

Thymuses from mice with 3, 12 and 24 months of age (n = 3 for age group) were obtained after perfusion with phosphate buffer saline and heparin, followed by 4% paraformaldehyde (Sigma-Aldrich, St Louis, MO, USA). Thymic lobes were separated. One of the lobes was maintained intact while the other was fragmented in approximately 1mm pieces. Both were fixed using 4% paraformaldehyde for 48h and 2.5% glutaraldehyde (EMS, Hatfield, PA, USA) in 0.1M sodium cacodylate buffer solution (PBS) for 2 h. Next, samples were washed in the same buffer (3 x 10 min) and post-fixed with buffered 1% osmium tetroxide (TedPella Inc., Redding, CA, USA) for 1h, before dehydration in graded series of acetone and embedding in Epon (Poly/Bed 812) (Polyscience Inc., Warrington, PA, USA) or low viscosity Spurr resin (Ted Pella Inc.). Blocks were incubated at 70°C for 24h for resin polymerization. Semi-thin and ultra-thin tissue sections were obtained using glass and diamond knives (Diatome, Hatfield, PA, USA), respectively, using a Power Tome X ultramicrotome (Boeckeler Instruments, Tucson, AZ, USA). Ultra-thin sections were stained with 2% uranyl acetate and 1% lead citrate and examined in a Zeiss EM900 transmission electron microscope (Zeiss, Oberkochen, BW, Germany) operated at 80 kV.

### Histotechniques for cellular lipid droplets visualization

Thymic semi-thin sections used for TEM were counterstained with toluidine blue (Sigma-Aldrich; C.I.: 52040). The osmium tetroxide fixation allowed the visualization of lipid droplets with yellow-brownish color. In order to visualize lipid droplets of thymic cells in frozen thymus sections, we fixed samples in 4% paraformaldehyde buffered solution for 12 hours before incubation with graded series of saccharose/saline solution until 30%. After, thymus samples were embedded in Tissue Tek® O.C.T. resin (Sakura, Torrance, CA, USA) and cut in 5μm thick using a cryostat (Leica CM 1850, Wetzlar, HE, Germany). Tissue sections on glass slides were fixed in ice-cold acetone and kept at -20°C. For lipid staining, samples were washed with distilled water and then 100% propylene glycol before incubation in a solution of 7% Oil Red O dye (Sigma-Aldrich, C.I.: 26125) in propylene glycol (Reagen, Colombo, Brazil) for 1h. Tissue sections were washed in 85% propylene glycol and water before counterstaining with toluidine blue. Following washing in water, glass slides with tissue samples were mounted in an aqueous media with 50% glycerol (Sigma-Aldrich) and 50% PBS (pH 8.5). Photomicrographs were recorded using an Axioplan microscope equipped with a 5.0MP camera (Zeiss).

### Quantification of fresh thymocytes and intrathymic LLMC and mast cells in situ

Fresh thymocytes were obtained using a Potter glass homogenizer. Cell suspension was centrifuged at 4°C for 5 min and ressuspended in 1mL of PBS with 5% fetal bovine serum (Gibco, Grand Island, NY, USA). Thymocyte cellularity was evaluated by counting using a hemocytometer.

Thymic tissue semi-thin sections of young (2 to 3 month-old), middle-aged (12 to 13 month-old) and old (22–24 month-old) mice were used for LLMC and mast cell quantification (n = 4, for young and middle-aged mice; n = 3, for old mice).

We performed histomorphometric analysis to evaluate the density of total LLMC in the thymus of distinct age ranges, considering the LLMC located deeper in the parenchyma (P) or in the subcapsular (SC) or perivascular parenchymal (PV) areas. LLMC density was verified quantifying their number per tissue area (mm^2^) of thymus sections stained for neutral lipids, with either osmium tetroxide solution or Oil Red O dye, followed by counterstaining with toluidine blue dye. Similarly, the density of mast cells in the aging thymus tissue section was analyzed following their identification through metachromasia with toluidine blue dye. The ImageJ software (Rasband, W.S., National Institutes of Health, Bethesda, Maryland, USA, http://rsb.info.nih.gov/ij/, 1997–2012) was used to quantify cells and the area of thymic tissue sections.

### Immunohistochemistry

Frozen thymic tissue sections were washed with PBS and incubated with potassium permanganate 0.06%, to reduce autofluorescence of lipid droplets [21]. Tissue samples were washed, incubated with ammonium chloride 50mM and washed twice with PBS before incubation with 1% bovine serum albumin diluted in PBS (Sigma-Aldrich) for 30 min. Tissue samples were incubated with the following primary antibodies: rabbit anti-mouse Iba1 polyclonal (019–19741, Wako, Chuo-Ku, Osaka, Japan); rabbit anti-mouse PPARγ2 (PA1-824, ABR Affinity Bioreagents, Golden, CO, USA); rabbit anti-mouse UCP-1 (UCP11-S Alpha Diagnostics International, San Antonio, TX, USA); rabbit anti-mouse NG2 polyclonal (sc20162, Santa Cruz, Dallas, TX, USA); rabbit anti-human α-SMA polyclonal (ab5694, Abcam, Cambridge, CA, UK); rabbit anti-bovine pan-cytokeratin polyclonal (Z0622, Dako, Glostrup, Denmark); or rabbit anti-Beclin1 polyclonal (ab62472, Abcam, Cambridge, CA, UK), at 1:100 dilution for 16h at -4°C. Samples were washed with PBS before incubation with donkey anti-rabbit IgG Alexa 488 secondary antibody (A-21206, Invitrogen, Grand Island, NY, USA), diluted 1:400 for 30 min at 37°C or 2h at room temperature, and then washed with PBS. Thymic tissue sections were incubated for 2h at room temperature with the polyclonal goat Ab against human perilipin (N-14; sc47322, Santa Cruz), that cross-react with mouse, diluted 1:100. After washing with PBS, slides were incubated with Donkey Anti-Goat IgG conjugated to Alexa 555 (A-21432, Invitrogen, Grand Island, NY, USA) diluted 1:500 for 1h at room temperature. After staining with DAPI (2μg/ml) (D1308 Invitrogen, Grand Island, NY, USA) or Hoechst (1μg/ml) (H1399, Invitrogen, Grand Island, NY, USA) for 10 min, slides with thymus tissues were mounted using SHUR/Mount media (Ted Pella Inc.). Photomicrographs were obtained using an Olympus LX-17 fluorescence microscope (Olympus Corporation, Shinjuku-ku, Tokyo, Japan) or a Zeiss Axio Observer Z1 microscope equipped with an ApoTome attachment

### Statistics

Results were considered statistically significant when P≤ 0.05, following one-way ANOVA with Newman-Keuls post-test analyzed using GraphPad Prism version 5 for Windows (GraphPad Software, La Jolla, CA, USA). For thymocyte cellularity, results are shown as mean ± standard error of the mean (SEM).

## Results

### LLMC present morphological variation and their density increases in distinct areas of the thymic parenchyma with age

**Fig 1:**
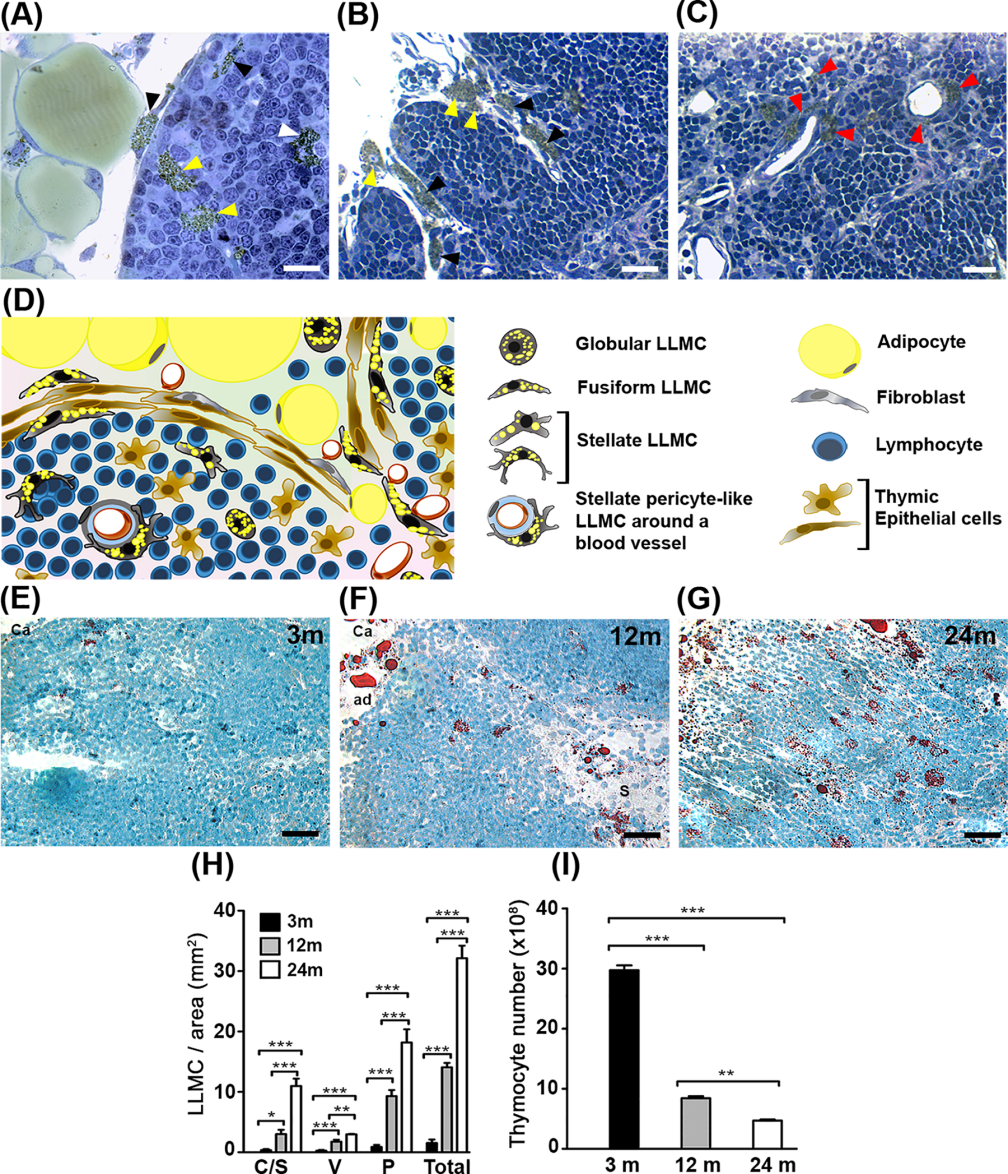
Distribution, morphology and density analysis of LLMC in the aging thymus. Semi-thin thymic section of middle-aged mouse shows LLMC with brownish yellow color due to fixation with osmium-tetroxide. **A:** intrathymic LLMC presenting fusiform (black arrowhead), globular (yellow arrowhead) or stellate (white arrowhead) morphologies in the thymic parenchyma. **B:** LLMC adjacent to the thymic septa with fusiform (black arrowheads) and globular (yellow arrowhead) morphologies. **C:** Fusiform or Stellate pericyte-like LLMC (red arrowhead) visualized around vessels in the thymic parenchyma, adjacent to endothelial cells. **D**: Schematic representation showing distribution and morphologies of LLMC in the aged thymus. **E, F** and **G**: Oil Red O lipid staining of thymic cryosections from mice with 3, 12 and 24 months of age, respectively. ad: adipocyte; Ca: capsule. Scale bars in A: 20μm; B and C: 25μm; D, E, F: 30μm; **H**: Distribution and density of LLMC in the whole area of 5μm thymic tissue sections from mice with 3, 12 or 24 months of age (n = 4). C/S represents SC-LLMC adjacent to capsule or septa; V represents PV-Perivascular LLMC; and P represents P-LLMC, deeper in the parenchyma. **I**: Total lymphocyte number in the thymus of mice with 3, 12 or 24 months of age (n = 4, per each age group). Results are shown as mean ± SEM. *p<0.05; **p<0.005; ***p<0.0005.

To investigate the morphology of LLMC in the aging thymus, we performed histological analyses in tissue sections of mice with 3 (young), 12 (middle-aged) and ~22–24 (old) months of age. Although rare in the young and better observed in the thymus of aged mice, we found LLMC in distinct areas of the thymic parenchyma, where these cells presented morphological variation, including globular, fusiform shape, like fibroblastoid cells, or stellate shape, also around vessels embracing thymocytes (Fig 1).

To evaluate LLMC considering their distribution in distinct areas of the thymic parenchyma, we classified all LLMC find deeper in the parenchyma as P-LLMC (Fig 1A) due to the difficulty to demarcate the limit between cortex and medullary regions in various thymus tissue sections of middle-aged and old mice. In addition, the LLMC localized adjacent to the capsule or septa regions, composed of connective and adipose tissues were called subcapsular LLMC (SC-LLMC) (Fig 1A and 1B) and those adjacent to blood vessels in the parenchyma were termed perivascular LLMC (PV-LLMC) (Fig 1C). While SC-LLMC showed globular or fusiform shape, like fibroblastoid cells, P-LLMC and PV-LLMC varied from globular to fusiform or stellate shape, with various protrusions between thymic lymphocytes and/or stromal cells (Fig 1). The different morphologies and distribution of intrathymic LLMC are schematically shown in Fig 1D.

The quantification of intrathymic LLMC in mice during aging evidenced few of these cells already present in the thymus of young mice. However, the density of these cells increases significantly from young to middle age and to old age (Fig 1E, 1F, 1G and 1H). When comparing the older with the youngest animals, the increase in the density of P-LLMC was followed by that in the SC-LLMC and PV-LLMC (Fig 1). On the other hand, we found a decrease of 68.3% in the number of thymic lymphocytes from young to middle aged; of 84.2% from young to old mice and of 44.3% in the number of thymocytes from middle-aged to old mice (Fig 1I). Taken together, there is a clear correlation between the increase of intrathymic LLMC density and the age-associated thymic involution process.

### LLMC distributed in distinct areas of the thymic parenchyma may interact with lymphocytes and stromal cells

**Fig 2:**
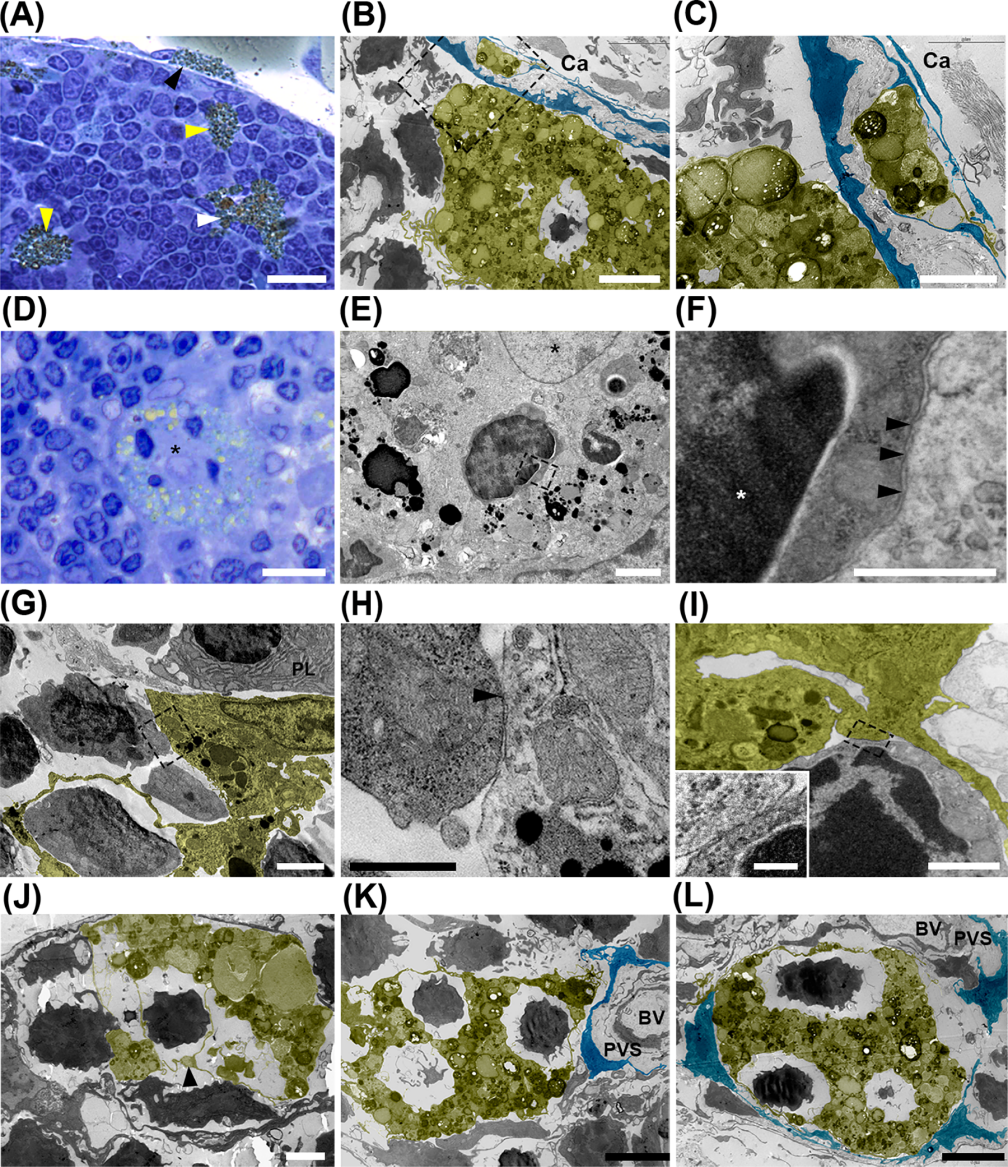
Intrathymic LLMC possibly interact with lymphocytes, epithelial and endothelial cells. **A:** Representative photomicrograph of fusiform SC-LLMC (black arrowhead); and globular (yellow arrowhead) or stellate (white arrowhead) P-LLMC in middle-aged mouse thymus section. Tissue fixation with osmium tetroxide reveals lipid-droplets in brownish yellow color. **B:** TEM photomicrographs show P-LLMC (false colored yellow), adjacent to subcapsular epithelial cells (SCE) (false colored blue), possibly interacting with lymphocytes in the thymic parenchyma. Selected traced area show SCE and LLMC in the limitation between parenchyma and capsule region (Ca); **C:** Higher magnification of the selected area in panel B shows a LLMC prolongation (false colored yellow) in the basal membrane between the two layers of SCE (false colored blue), in the limit of the thymic parenchyma. **D**: Semi-thin thymus tissue sections fixed with osmium tetroxide and counterstained with toluidine blue reveal globular P-LLMC (black asterisk) surrounding four lymphocytes. **E:** TEM photomicrograph showing a lymphocyte surrounded by the P-LLMC in Panel D. Selected traced area shows limiting areas between lymphocytes and LLMC (black asterisk in the nucleus); **F**: Higher magnification of the selected area in panel E shows the nucleus (white asterisk) and plasma membrane of a lymphocyte (black arrowheads) surrounded by the LLMC with a more electron-lucent cytoplasm. **G:** A LLMC (false colored yellow) embraces a lymphocyte with a telopode-like prolongation and possibly establishes membrane contact with another lymphocyte in the old thymus. A plasma cell (PL) is located adjacent to an intrathymic LLMC. **H:** Higher magnification of selected area in G shows possible membrane contact between LLMC and lymphocyte. Black arrowhead indicates close apposition between cell membranes; **I:** A P-LLMC (false colored yellow) possibly establishing contact with a lymphocyte. Inset on the bottom left corner demonstrates higher magnification of selected area showing close apposition between the cell membranes of LLMC and lymphocyte; **J:** P-LLMC (false colored yellow) showing a telopode-like prolongation embracing thymic lymphocytes. Telocyte podom-like structure is indicated (black arrowhead). **K:** TEM photomicrograph showing P-LLMC (false colored yellow) surrounding lymphocytes close to thymic epithelial cell (false colored blue) around the perivascular space (PVS); **L:** TEM photomicrograph showing LLMC (false colored yellow) in the PVS surrounded by epithelial cells (false colored blue). BV, blood vessel. Ca: capsule; PL: plasma cell; PVS: Perivascular space. Scale bars in A: 20 μm; B, G, J, L: 5 μm; C, E, K: 2 μm; D: 15 μm; F,H: 0.5 μm; I: 1μm and inset in I: 0.2 μm.

To analyze LLMC interaction with lymphocytes and other microenvironmental cells in the aging thymus, we initially used light microscopy and we found LLMC embracing lymphocytes and/or potentially interacting with lymphocytes in distinct regions of the aging thymus (Fig 2A and 2D). By TEM, we observed SC-LLMC surrounding lymphocytes close to the layer of subcapsular epithelial cells that delineate the thymic parenchyma (Fig 2B). In this regard, it is known that one or two layers of epithelial cells are present in the subcapsular region of the thymus [22]. We observed part of a LLMC in the basal membrane between the layers of epithelial cells (Fig 2C), suggesting that the LLMC could possibly be migrating into the thymus from the capsule of connective tissue. By light microscopy and TEM, we could also identify globular P-LLMC surrounding thymocytes (Fig 2D, 2E and 2F). This LLMC presented a central euchromatic nucleus and numerous lipid droplets peripherally distributed. In higher magnification, we verified the P-LLMC embracing thymocytes (Fig 2E and 2F). We should mention that lysosomes were not visualized in the cytoplasmic area of the P-LLMC involving the lymphocyte, suggesting that this cell was not being phagocytized by the P-LLMC (Fig 2F). The membranes of both P-LLMC and lymphocyte were clearly observed (Fig 2F). In addition, we found P-LLMC embracing thymic lymphocytes through long thin structures (Fig 2G), resembling telocytes with long thin monoliform prolongations [23]. We noticed close apposition between P-LLMC and thymic lymphocytes (Fig 2G, 2H and 2I). We also observed, next to a telocyte-like P-LLMC, a plasma cell with darker regions of heterochromatin in the periphery of the nucleus and a well-developed granular endoplasmic reticulum, thus indicating protein synthesis activity. Telocyte-like P-LLMC presented intermediate dilated areas of cytoplasm, like podom structures (Fig 2J). The spaces left between the thin membrane projections of the P-LLMC and lymphocytes (Fig 2J) could likely form a selective niche in the thymic microenvironment, where these cells could interact in a paracrine manner, for instance, through soluble factors signaling. In addition, we observed a P-LLMC with cytoplasmic projections embracing thymocytes, close to epithelial cells surrounding the perivascular space of blood vessels (Fig 2K). LLMC embracing lymphocytes were also identified in the perivascular space surrounded by thymic epithelial cells (Fig 2L).

**Fig 3:**
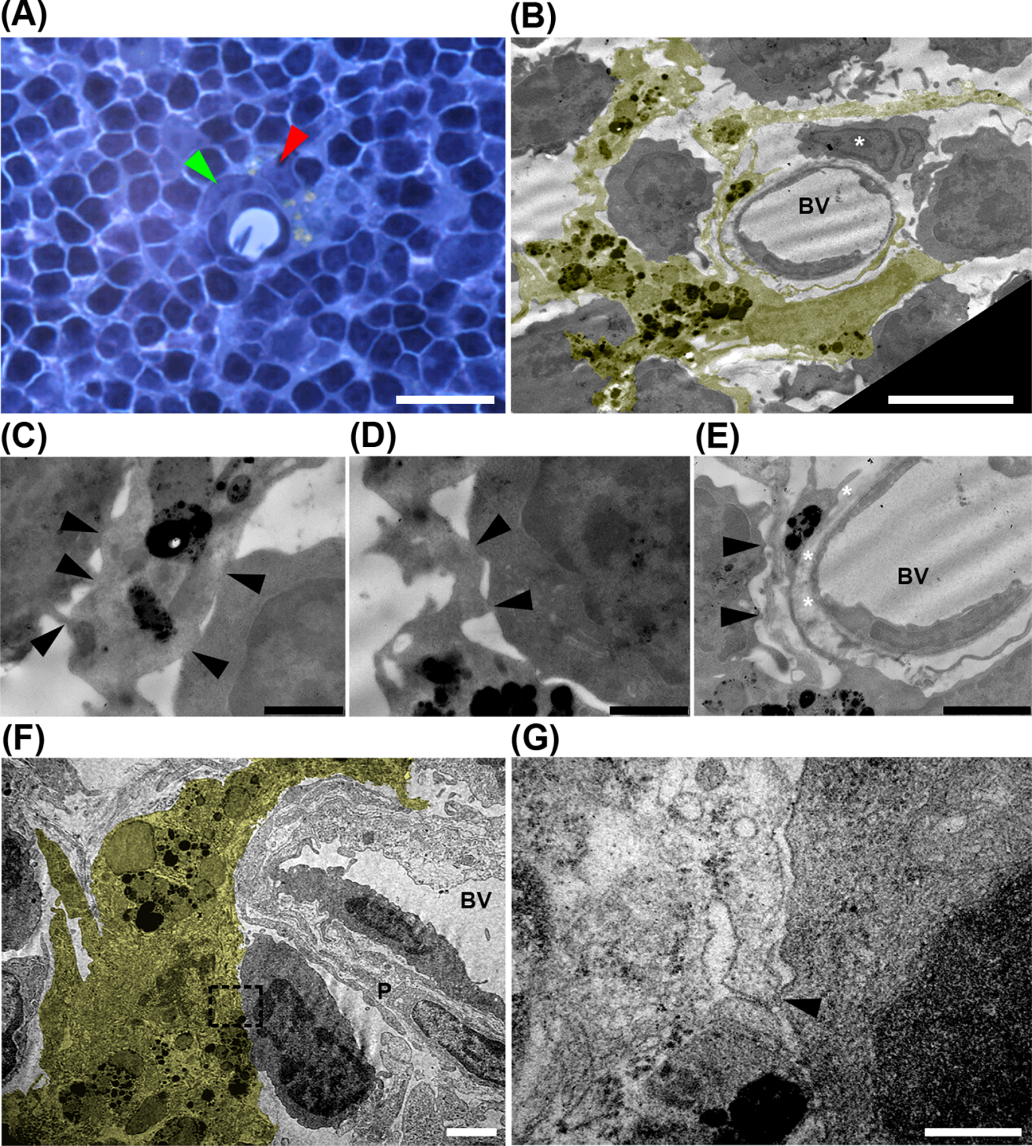
Pericyte-like LLMC possibly interacting with lymphocytes and endothelial cells in the aging thymus. **A:** Semi-thin thymic section of middle-aged mice fixed with osmium tetroxide and stained with toluidine blue shows pericyte (green arrowhead) and pericyte-like LLMC (red arrowhead) possibly interacting with endothelial cells. **B:** TEM photomicrograph shows pericyte-like LLMC (false colored yellow) surrounding an endothelial cell and possibly interacting with various lymphocytes. White asterisk indicates elongated nucleus of a migrating lymphocyte. **C-E:** Higher magnifications of regions seen in B. Black arrowheads indicate close apposition between membranes of LLMC and thymic lymphocytes. In E, LLMC and endothelial cell share basal membrane. Asterisks indicate area of extracellular matrix that forms basal membrane **F:** PV-LLMC (false colored yellow) surrounding an endothelial cell and possibly interacting with lymphocytes in an old thymus. **G:** Higher magnification of selected area in F. Black arrowhead shows possible membrane contact between LLMC and lymphocyte. BV: blood vessel; P: pericyte. Scale bars in A: 15μm; B: 5μm; C, D: 1μm; and E: 2μm; F: 2μm and G: 400nm.

We found PV-LLMC possibly interacting with thymic endotelial cells and pericytes in aged mice (Fig 3A). Pericytes are known as mesenchymal cells that embrace endothelial cells and share with these cells the basement membrane [24]. Through TEM, we observed PV-LLMC with long cytoplasm projections possibly contacting endothelial cells and several thymic lymphocytes (Fig 3B, 3C, 3D, 3E, 3F and 3G). Because we have not find cytoskeleton filaments, the interaction between the PV-LLMC and lymphocytes could be composed by membrane proteins. For instance, integrins are used by thymocytes to bind to their corresponding ligand receptors on microenvironmental cells to migrate through the thymic parenchyma.

**Fig 4:**
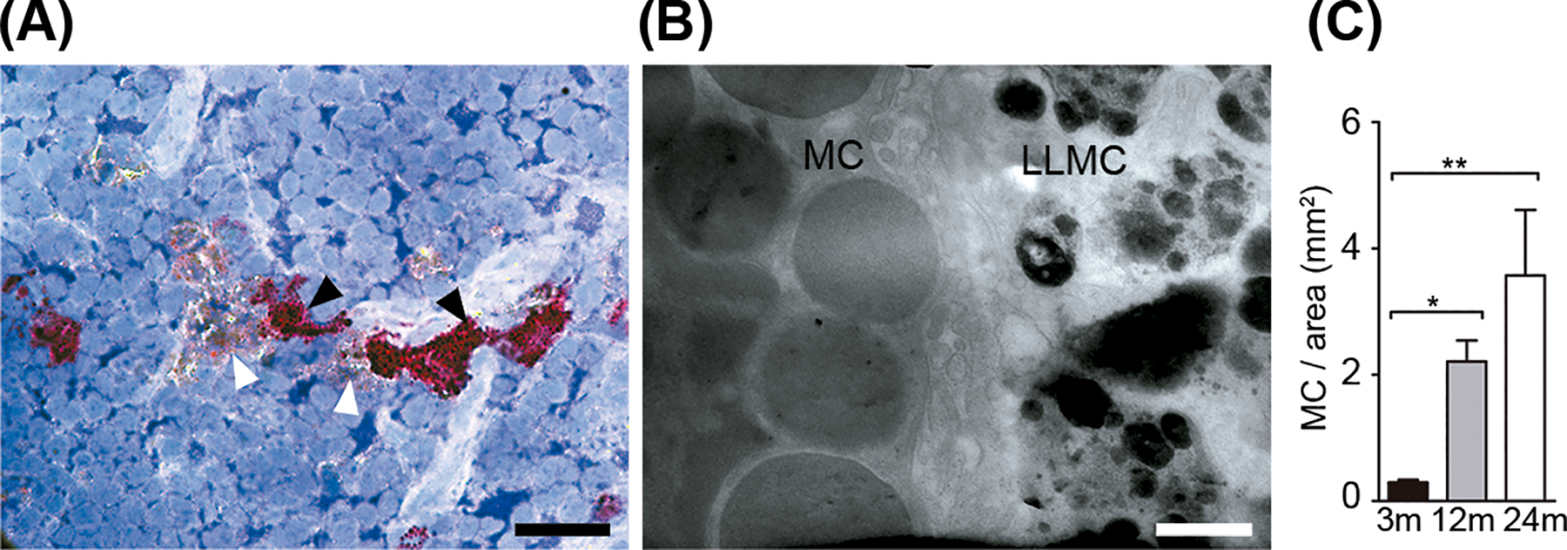
LLMC may interact with mast cells in the thymus. **A:** Oil Red O^+^ LLMC (white arrowheads) possibly interacting with toluidine blue-stained metachromatic purple mast cells in the thymus from middle-aged mice. **B:** TEM photomicrograph shows intrathymic LLMC contacting mast cell (MC). Scale bars in A: 15 μm; and B: 0.5 μm. **C:** Graphic representation of intrathymic mast cells density in mice with 3, 12 or 24 months of age (n = 4). Results are shown as mean ± SEM. * p<0.05; ** p<0.005.

Intrathymic P-LLMC positively stained with Oil Red O, a marker for neutral lipids, localized close to purple metachromatic mast cells stained with toluidine blue dye (Fig 4A). Through TEM, we visualized thymic mast cell with many cytoplasmic granules adjacent to P-LLMC, suggesting that these cells may establish direct contact with each other (Fig 4B). We verified that the density of thymic mast cells increases progressively with age (Fig 4C).

These data demonstrate LLMC as a thymic microenvironmental component possibly capable of interacting with lymphocytes and stromal cells, such as epithelial cells and pericytes as well as mast cells, at least through cell-cell membrane contacts.

### LLMC present autolipophagosome-like structures and autophagic activity

**Fig 5:**
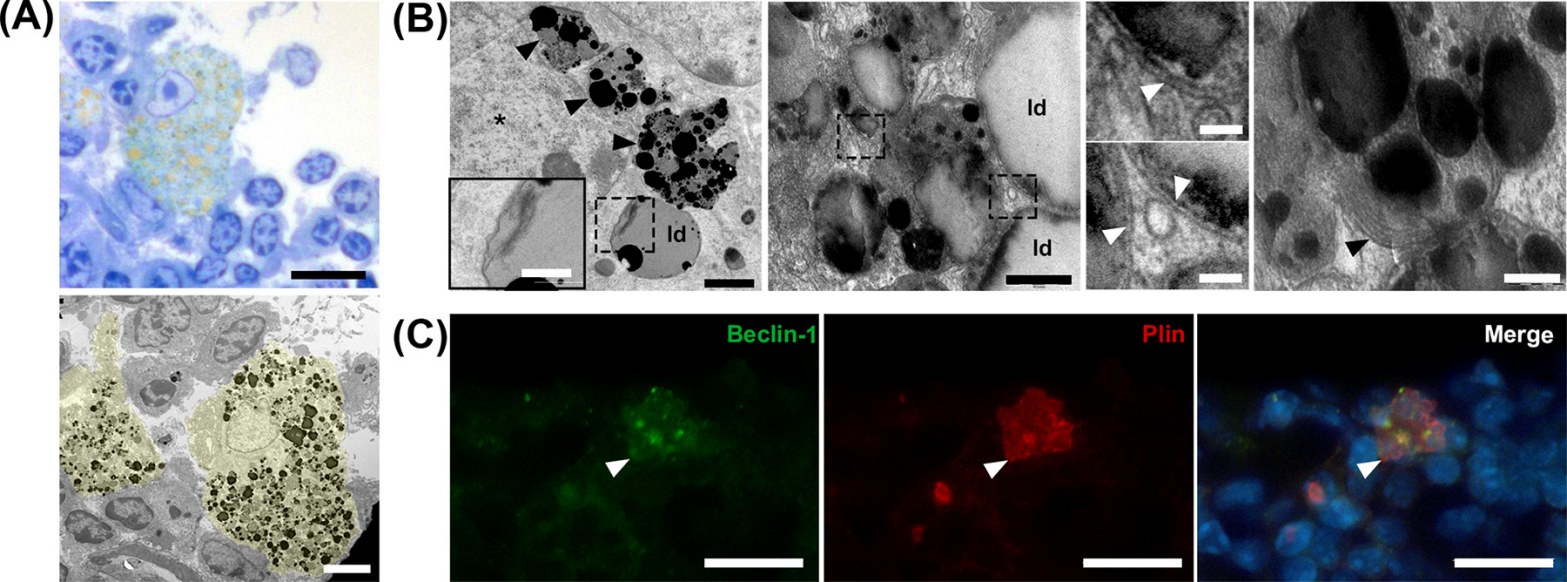
Intrathymic LLMC present lipid droplets and vesicles of different electrondensities and autolipophagossome-like structures. **A:** Upper panel shows intrathymic LLMC in middle-aged mouse. Semi-thin section fixed with osmium tetroxide. LLMC stained in brownish yellow. Lower panel shows TEM higher magnification of LLMC (false colored yellow) seen in the upper panel containing vesicles of different electrondensities. **B:** Left panel shows TEM photomicrograph of cytoplasmic lysosomes (indicated by black arrowheads) with lipofuscin and mixed content next to the LLMC nucleus (indicated by a black asterisk). Selected area with autolipophagosome-like multimembrane structure inside a lipid droplet is shown in higher magnification in the inset on the bottom left corner. Middle panel shows vesicles and lipid droplets of LLMC presenting electron-dense membranes typical of autolipophagosome formation. Small panels correspond to higher magnification of selected areas in the middle panel. White arrowheads indicate double membranes of autophagossomes with mixed content in lipid droplets. Right panel shows cytoplasmic lipofuscin granules and distinct electron-dense lipid droplets surrounded by a multi-laminar structure (indicated by a black arrowhead) in a LLMC. **C:** intrathymic LLMC (white arrow) positively labeled for Beclin-1 (green) and PLIN (red) by immunofluorescence. Cell nuclei labeled with Hoechst (blue). ld: lipid droplets. Scale bars in A, upper panel: 15μm, lower panel: 4μm; B left panel: 1μm, inset: 0.5μm; in B middle panel: 0.5μm; in B small panels: 0.1μm; in B right panel: 0.2μm; C: 15 μm.

We further examined the ultrastructure of LLMC (Fig 5). The yellowish lipid droplets observed in LLMC stained with osmium tetroxide, by light microscopy, corresponded to lipid droplets with different electrondensities, as analyzed by TEM (Fig 5A). In this regard, osmium tetroxide used in tissue preparation has high affinity for unsaturated lipids indicating that the darker droplets contain more unsaturated lipids than the lucent droplets [25]. The distinct osmiophilic profile in LLMC indicated a mixture of lipid content in these cells (Fig 5A and 5B). Moreover, we observed small membranous vesicles resembling lysosomes filled with lipids and lipofuscin granules in intrathymic LLMC (Fig 5B). Double membrane vesicles, characteristic of autophagosomes, with mixed content, lipofuscin or only lipids (resembling autolipophagosomes) were visualized in LLMC as well (Fig 5B). In this respect, Singh and colleagues [25] were the first to describe the characterization and formation of autolipophagossomes, which are lipid-containing double-membrane vesicles. Here, we observed multimembrane structures inside cytoplasmic lipid droplets, which suggests the formation of autolipophagossomes in thymic LLMC (Fig 5B).

Furthermore, we have used a marker for perilipin to analyze its possible co-expression with Beclin-1, an autophagosome formation molecule [26, 27], in intrathymic LLMC. We visualized some PLIN^+^Beclin-1^+^ LLMC in the thymic parenchyma (Fig 5C). Conjointly, our results strongly suggest that autophagic activity, including lipophagy, occur in intrathymic LLMC.

### Intrathymic LLMC are phenotypically heterogeneous

Because intrathymic LLMC phenotype is still not clearly defined, we sought to evaluate the phenotype of intrathymic LLMC analyzing *in situ* the expression of distinct molecules that characterizes lipid-accumulating cells, including markers for preadipocytes [13], brown adipocytes [28], foam macrophages [29], pericytes or mesenchymal stem cells [24, 30]. In addition, we analyzed whether thymic epithelial cells accumulate lipids in the aging thymus.

**Fig 6:**
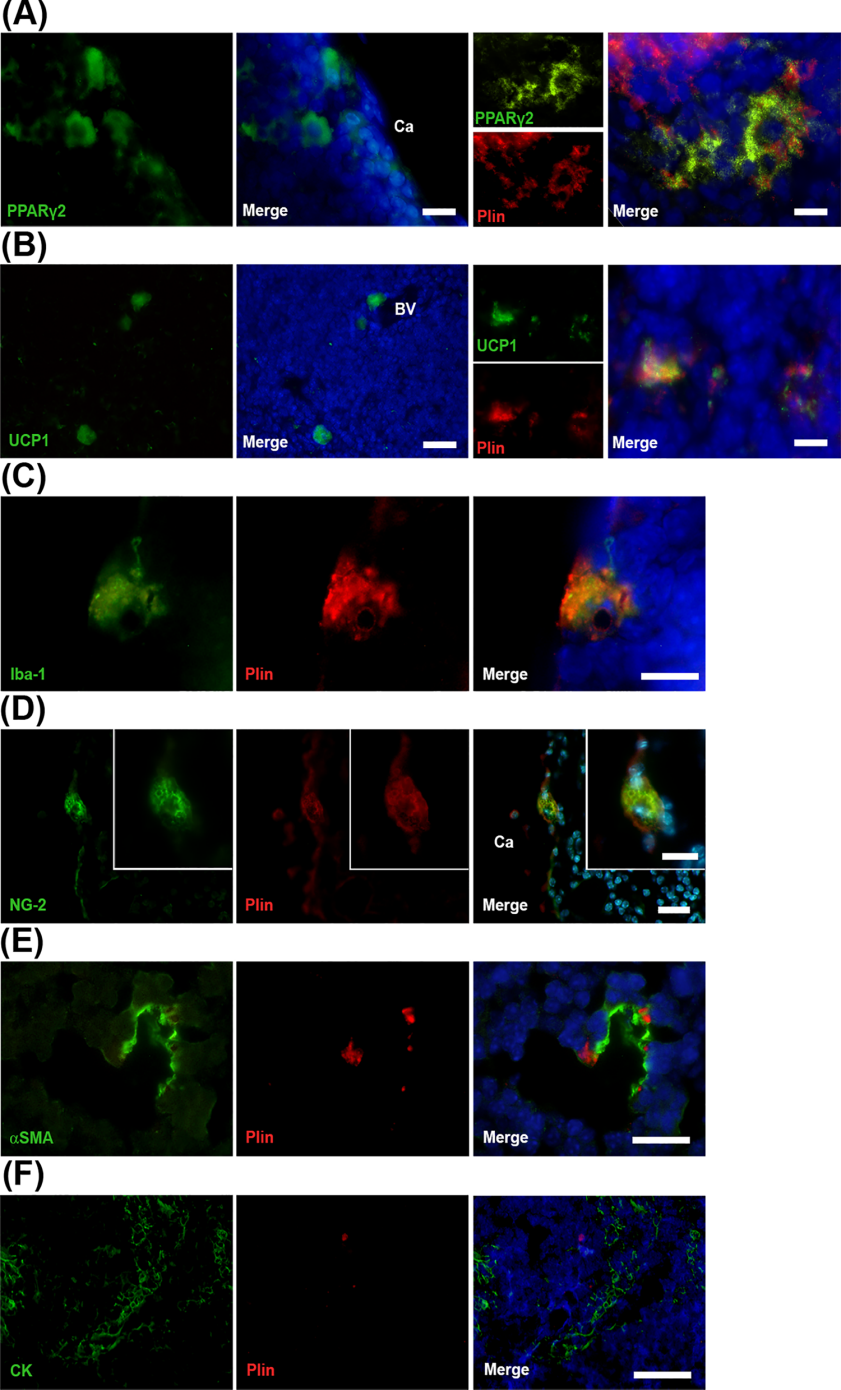
Heterogeneity of intrathymic LLMC phenotypes. **A**: Left panel: Preadipocytes immunostained for PPARγ2 in the thymic parenchyma close to the capsule and blood vessels. Small panels show representative photomicrographs of intrathymic lipid-laden preadipocytes immunolabeled for PPARγ2 (green) or PLIN (red); merged images in the right panel. **B:** Left panel shows thymic brown adipocytes expressing UCP-1 near blood vessels in the parenchyma. Small panels show representative photomicrographs of intrathymic brown adipocytes immunolabeled for UCP1 (green) or PLIN (red); merged images in the right panel **C:** intrathymic macrophagic LLMC expressing PLIN (red) and Iba 1 (green). **D:** Pericytic LLMC (white arrowheads) expressing PLIN (red) and NG2 (green) adjacent to the capsule. Insets show higher magnification of perilipin and NG2 staining around the lipid droplets of the pericytic LLMC. **E**: myofibroblastic LLMC stained with antibody for PLIN (red) and αSMA (green) surrounding a blood vessel. **F:** Thymic epithelial cells positively stained with antibody anti-Pan cytokeratin (CK, green) did not express PLIN (red). Nuclei stained with DAPI or Hoechst dye (blue). BV: blood vessels, Ca: capsule, S: septae. Scale bars in A, left panels: 15μm; right panel: 7 μm in B, left panels: 25μm; right panel: 5μm; in C: 10μm; in D: 20μm, inset: 10μm; in E: 15μm; in F: 30μm.

We found that SC-LLMC, P-LLMC and PV-LLMC were positively stained for PPARγ2 and UCP1, which are specific markers for the “professional” lipid-laden cells pre-adipocytes and brown adipocytes, respectively (Fig 6A and 6B). Although the anti-PLIN antibody did not label efficiently all lipid droplets as Osmium Tetroxide or Oil Red O dyes, it was possible to use it in combination with distinct cellular phenotype markers to, qualitatively, identify intrathymic LLMC by immunohistochemistry. Both PPARγ2^+^ and UCP1^+^ intrathymic cells expressed PLIN. Next, we evaluated the possible presence of lipid droplets in non-professional lipid-laden cells in the aging thymus. We found PLIN^+^LLMC expressing the Iba1 macrophage marker (Fig 6C). NG2^+^PLIN^+^ SC-LLMC were also observed in the thymus of aged mice, indicating that intrathymic LLMC correspond to the pericyte phenotype as well (Fig 6D). In addition, PV-LLMC were positively stained with antibodies against PLIN and smooth muscle alpha-actin (αSMA), a marker for myofibroblasts, smooth muscle cells and pericytes (Fig 6E). On the other hand, pan-cytokeratin^+^ thymic epithelial cells were negative for the expression of PLIN (Fig 6F). These data reveal that intrathymic LLMC comprise heterogeneous cellular phenotypes, including “professional” lipid storing cells (adipocytes), foam macrophages and distinct mesenchymal cell types accumulating lipids during the aging process.

## Discussion

The current study elucidates that intrathymic LLMC correspond to not only one but to various distinct cellular phenotypes, including preadipocytes, brown adipocytes, lipid-laden pericytes and foam macrophages. Intrathymic LLMC density increases with age. These cells are in close apposition to pericytes and endothelial cells. Moreover, frequent finding of the intermittent close apposition between LLMCs and lymphocytes indicates that LLMC have a contact with lymphocytes, suggesting active role for these cells in the aging thymus. Our data also bring new insights on the origin and functions of intrathymic LLMC under the influence of age-associated dyslipidemia, inflammation and oxidative stress processes. There are several ways to explain the presence of LLMC in the aged thymus.

A first possibility is that LLMC migrate into the thymus from the extrathymic adipose tissue. In this regard, migrating cells of the adipose tissue include preadipocytes and mesenchymal stromal cells [17–18]. In this context, we have previously shown the presence of LLMC in the perithymic adipose tissue and capsular region, as well as inside the thymic parenchyma, expressing the CCR5 chemokine receptor [18]. Their proximity to subcapsular epithelial cells and the increased expression of CCR5 proinflammatory ligands in the aging thymus first suggested that LLMC could be perithymic adipose tissue cells migrating into the thymic parenchyma [18]. Supporting this hypothesis, mesenchymal cells are able to migrate to the mouse thymus *in vivo* [30]. In addition, different cell types derived from mesenchymal lineage analyzed here are found in visceral adipose tissue under inflammatory conditions [31]. Thus, it is reasonable to consider that distinct types of LLMC could be migrating from the perithymic adipose tissue to the aging thymus parenchyma. Furthermore, we observed brown adipocytes in the subcapsular region of the aging thymus. As white adipose tissue, brown adipose tissue is present around the thymus of rodents [32]. Moreover, brown adipose tissue proteins were described to promote thymic atrophy [32]. Further studies are necessary to evaluate the role of brown adipocytes in the aging thymus.

A second possibility is that intrathymic LLMC could actually be thymic mesenchymal stromal cells accumulating lipids in response to the inflammatory environment and increase of oxidized LDL and oxidative stress that occurs in the thymus with age [33]. In this regard, Kirkland and colleagues have suggested that during the natural process of aging, dyslipidemia occurs and distinct mesenchymal cell types might acquire the profile of mesenchymal-adipocyte like default (MAD) cells [34], in association with the increase of pro-inflammatory molecules in circulation, also known as inflammaging [35]. Indeed, mesenchymal cells are known to be present inside the thymic anlage, where they play a crucial role for thymic development and function [36]. Moreover, pericytes share common features with mesenchymal stromal cells and are, therefore, also able to differentiate into distinct cell lineages according to the presence of adipogenic, osteogenic or chondrogenic factors [24, 30].

A third possibility to explain the origin of LLMC in the aging thymus is that intrathymic mesenchymal cells could possibly assume a macrophage phenotype that accumulates lipids in the aging thymus. While adipocytes originate from mesenchymal stromal cells in distinct tissues, macrophages are cells of bone marrow hematopoietic stem cell origin [37]. However, 3T3-L1 fetal mesenchymal cell line, which is the classic model to study preadipocyte differentiation to adipocytes, has been shown to assume macrophage characteristics and express macrophage markers in the peritoneal cavity of mice, showing its plasticity potential [38]. Additionally, in atherosclerosis, which is an age-associated pro-inflammatory condition, foam macrophages are known to produce higher amounts of TNF-α and IL-6 [39]. In this sense, foam macrophages could be actively involved in the basal inflammatory state associated with the thymic involution process. Furthermore, we do not discard the possibility that thymic dendritic cells could also be accumulating lipids in the aging thymus, as previously shown in age-unrelated inflammatory conditions [40].

We showed that thymic LLMC possibly interact with mast cells, which density increases in the thymus with age. The presence of mast cells in human thymus has been shown in pathological conditions, such as myasthenia gravis and thymoma [41]. Here, we did not find mast cells degranulating in the aging thymus. However, mast cells are described to be able of secreting pro-inflammatory cytokines and regulating macrophages activity without degranulation [16], which reinforce the hypothesis that foam macrophages might contribute for the basal inflammation in the aging thymus.

Curiously, we found LLMC as telocyte-like cells accumulating lipid droplets. Telocytes are morphologically described as stromal cells that present long projections, termed telopodes, with larger cytoplasmic areas containing organelles, called podoms [23]. These cells are genetically not similar to fibroblast or mesenchymal stem cells and they are described to play roles in tissue remodeling, cell signaling and angiogenesis [42]. Although the function of these cells in the aging thymus remains to be elucidated, we show that parenchymal telocytes like-cells accumulating lipids establish direct membrane contacts with lymphocytes.

Because our results did not show cytokeratin^+^ thymic epithelial cells expressing PLIN in the aging thymus, we suggest that thymic epithelial cells do not accumulate lipid droplets as LLMC during aging. We also did not find cytoskeleton filaments or tonofilaments in LLMC. However, we do not discard the possibility that epithelial cells could reduce cytokeratin expression before accumulating lipids, for instance. In this context, it has been suggested that thymic epithelium could transdifferentiate into preadipocytes or that epithelial to mesenchymal transition (EMT) and preadipocyte differentiation could occur in the aging thymus [19, 20]. Indeed, EMT can occur in organ fibrosis [43], which could be the case in the aging thymus, where ongoing extracellular matrix and tissue remodeling possibly contributes for a frustrated wound healing response to the age-related tissue degenerative process associated with basal inflammation. In this regard, although epithelial tissues are known to derive from all three embryonic germ layers, the thymic cortical and medullary epithelial cells derive from the endoderm [44]. As mesenchymal cells and macrophages, endothelial cells derive from the mesoderm and Frid and colleagues have shown endothelial-mesenchymal cell transdifferentiation *in vitro* [45]. However, whether endothelial cells transdifferentiate in LLMC in the aging thymus remains to be elucidated.

A fifth possibility is that intrathymic LLMC could be stressed cells with their autophagic machinery blocked, trying to survive in the aging thymus, as occurs under some conditions of excess of lipids. In this regard, inflammaging and increased oxidative stress have been associated with activation of inflammasomes by danger signals, such as cellular stress and induction of cellular autophagy [46]. In this sense, our analyses of intrathymic LLMC revealed the presence of lipofuscin, which is a feature of age-associated cellular stress [47]. Moreover, we observed that LLMC contain distinct lipids, since lipid droplets showed different electrondensities. We also found double- and multi-membrane structures in lipid droplets of distinct intrathymic LLMC. Intrathymic PLIN^+^ LLMC were positively stained for Beclin-1 marker, which confirmed autophagic activity in these cells. The multi-membrane structures in lipid droplets of LLMC resembled autolipophagosome formation observed by Singh and colleagues [26]. The authors suggested that autophagy is a basal mechanism for lipid metabolism and that autolipophagy could be a primary mechanism for cell survival in stress conditions [26].

Our findings show distinct phenotypes of LLMC as thymic components, which possibly interact with lymphocytes and other cells. These LLMC might have a panoply of actions regulating the physiology of the aging thymus. Future studies on the functional role of distinct LLMC may contribute to further understanding the molecular and cellular mechanisms regulating the age-related degenerative process of the thymus and for the development of potential regenerative therapies.
